# Sweat gland organoids contribute to cutaneous wound healing and sweat gland regeneration

**DOI:** 10.1038/s41419-019-1485-5

**Published:** 2019-03-11

**Authors:** Jinmei Diao, Juan Liu, Shuyong Wang, Mingyang Chang, Xuan Wang, Baolin Guo, Qunfang Yu, Fang Yan, Yuxin Su, Yunfang Wang

**Affiliations:** Stem Cell and Tissue Engineering Lab, Institute of Health Service and Transfusion Medicine, 100850 Beijing, China

## Abstract

Sweat glands perform a vital thermoregulatory function in mammals. Like other skin components, they originate from epidermal progenitors. However, they have low regenerative potential in response to injury. We have established a sweat gland culture and expansion method using 3D organoids cultures. The epithelial cells derived from sweat glands in dermis of adult mouse paw pads were embedded into Matrigel and formed sweat gland organoids (SGOs). These organoids maintained remarkable stem cell features and demonstrated differentiation capacity to give rise to either sweat gland cells (SGCs) or epidermal cells. Moreover, the bipotent SGO-derived cells could be induced into stratified epidermis structures at the air−liquid interface culture in a medium tailored for skin epidermal cells in vitro. The SGCs embedded in Matrigel tailored for sweat glands formed epithelial organoids, which expressed sweat-gland-specific markers, such as cytokeratin (CK) 18 and CK19, aquaporin (AQP) 5 and αATP. More importantly, they had potential of regeneration of epidermis and sweat gland when they were transplanted into the mouse back wound and claw pad with sweat gland injury, respectively. In summary, we established and optimized culture conditions for effective generation of mouse SGOs. These cells are candidates to restore impaired sweat gland tissue as well as to improve cutaneous skin regeneration.

## Introduction

Sweat glands, vital traits of skin, perform several primary functions including secretion of sweat, excretion of wastes, maintenance of body temperature and inhibition of bacterial growth by secretion of lactate^1,2^. However, sweat glands have limited ability to regenerate after full-thickness damage as that occurs with deep burns^3–5^. To date, there is no effective treatment available for patients with irreversible loss of functional sweat glands. The regeneration of a fully functional skin comprised of not only epidermis and dermis but also skin components, especially sweat glands, is a subject of great interest in clinical therapy. The key to combat this obstacle is to isolate appropriate sweat gland cells (SGCs) that can be used for sweat glands reconstruction.

The studies about sweat glands are not as clear as about other cutaneous components such as hair follicles and mammary glands. In addition, the SGCs are scattered in the dermis and difficult to harvest. Several studies reported that other types of cells have proved capable of differentiating into SGCs, including keratinocytes^6^, mesenchymal stem cells^7–9^, amniotic fluid-derived stem cells^10^, embryonic stem cells^11^, and induced pluripotent stem cells, etc. Nevertheless, these sources of cells are associated with low differentiation efficiency that limits the further application of these methods. Therefore, the important task in regeneration of skin with sweat glands is how to isolate SGCs on a large scale to establish skin with sweat glands.

Stem cells are the candidate resource for tissue regeneration, and previous studies have illustrated that the adult human sweat gland myoepithelial cell subpopulations contain stem cells that possess both self-renewal ability and multipotency that includes differentiation into sweat glands^12–14^. However, studies to date of isolated sweat gland stem/progenitor cells subjected to traditional monolayer culture always rapidly differentiated into keratinocytes and lost their specific phenotypic characteristics^3,15^. This implicates interactions among multiple cell types, extracellular matrix and growth factors as playing key roles in the development and characteristic maintenance of sweat glands^16^. Many studies have demonstrated that three-dimensional (3D) cultures, such as organoids, can re-establish these interactions and recapitulate the phenotypic traits of normal tissues, including for brain^17,18^, intestine^19–21^, liver^22,23^, pancreas^24,25^, prostate^26^, and so on. Lei et al. used the skin organoids to analyze tissue-level phase transition during the hair regeneration, demonstrating the this in vitro self-organization process achieved a similar phenotype in vivo^27^. During the process of organoid formation, the culturing medium containing growth factors can regulate the organoid-forming efficiency, the phenotypic traits of the organoids, and the longevity of the cultures. Therefore, development of a 3D organoid culture strategy for sweat glands may be able to maintain the specific characteristics of SGCs and achieve the enrichment and amplification of sweat gland stem/progenitor cells.

**Fig. 1:**
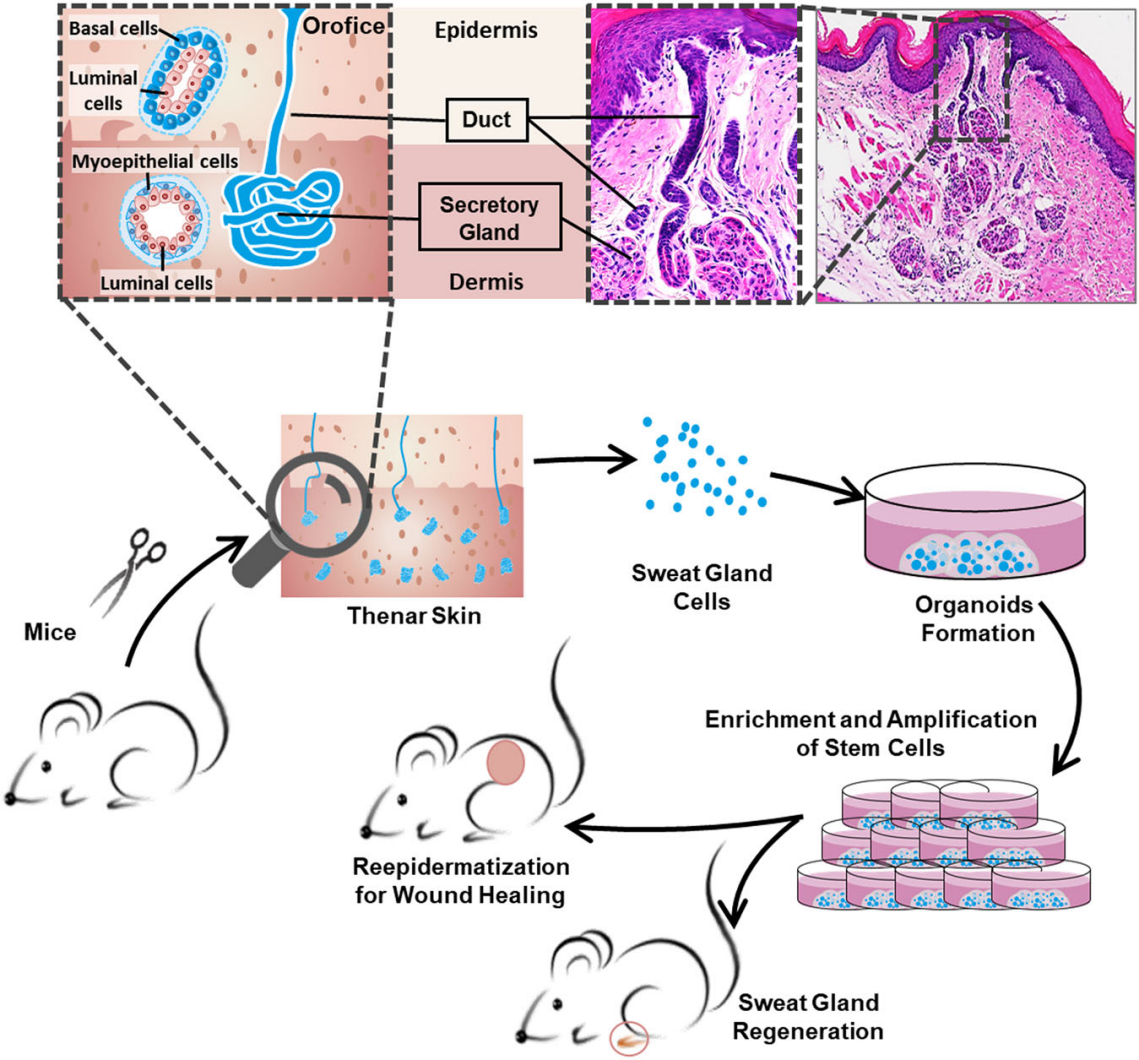
Schematic representation of the study. The sweat gland-derived stem cells were used for sweat gland regeneration and restoration of the epidermis in wound healing. The inside diagram showing orifice and intraepidermal portion of a sweat duct, which extends from epidermis into dermis and terminates in a coiled, secretory gland

Matrigel, a solubilized basement membrane preparation that contains laminin, fetal collagens, heparan sulfate proteoglycans, entactin, and containing many matrix-bound growth factors, has been found to help cells growing as organoids^28^. In this study, we established a systematic isolation procedure for mouse SGCs using an enzymatic digestion method and performed extensive work focusing on culture conditions of sweat gland organoid (SGO) cultures utilizing Matrigel (Fig. 1). The optimized culture conditions were able to successfully generate the SGOs with vigorous expansion capacity. More importantly, the sweat gland stem cells in the generated organoids maintained bipotency to lineage restrict either to sweat glands or epidermis, which should facilitate the wound-healing process and induce the in situ regeneration of sweat-gland-like structures in the skin. Results of this study provide an experimental basis for skin tissue engineering, especially skin with its various components such as sweat glands.

## Results

### Molecular profiling of mouse epidermis and sweat gland compartments

**Fig. 2:**
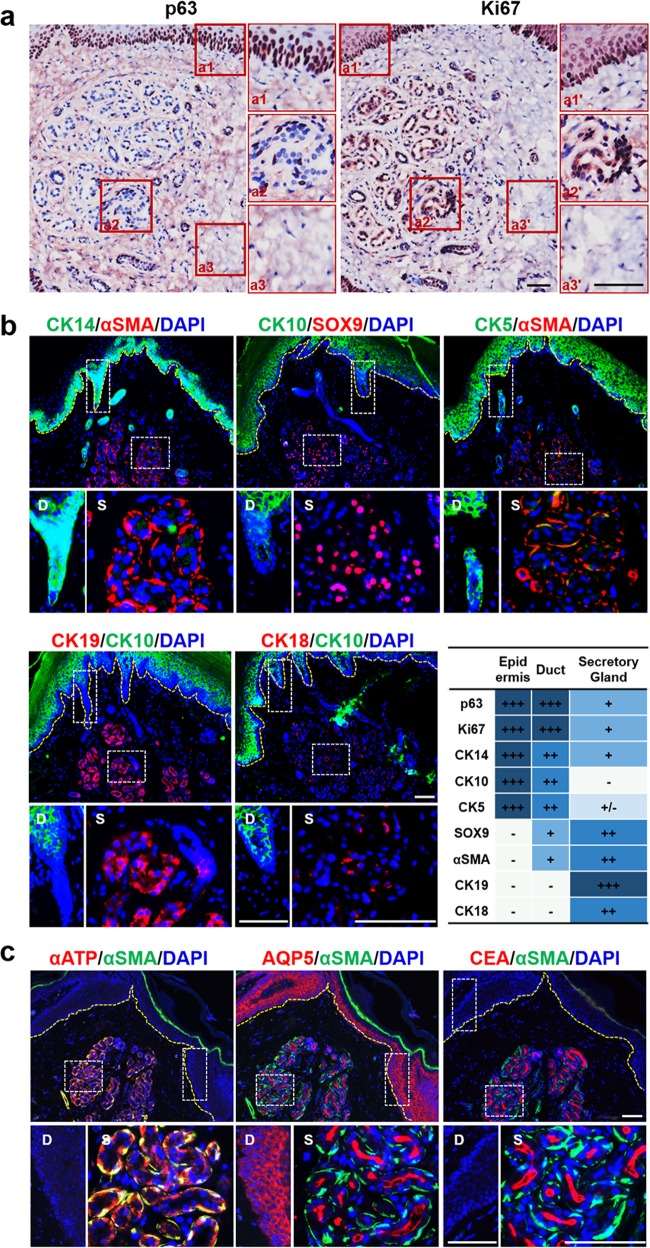
Histological staining revealed the expression of markers specific for epidermis and sweat glands in the adult mouse paw skin. **a** Immunohistochemical (IHC) staining of the basal/progenitor marker p63 and cell proliferation marker Ki67. The right images are magnifications of the boxes in the left image. The cells located at the basement membrane (a1, a1′) and sweat gland (a2, a2′) highly expressed p63 and Ki67. And the cells located at skin dermis (a3, a3′) did not express p63 and Ki67. **b** Immunofluorescence (IF) staining displayed the colocalization of epidermal cell markers (CK14, CK10, and CK5) with sweat gland cell markers (αSMA, SOX9, CK18, and CK19). The yellow dashed line represents the boundary between epidermis and dermis. The lower images show the respective magnification of the boxes in the upper images of the duct-connected epidermis (left) and the secretory gland under the deep dermis (right). The expression levels are summarized in the table. **c** Colocalization of sweat gland functional markers, αATP, AQP5, and CEA, with αSMA especially in the secretory portion of the sweat gland. Nuclei (blue) were counterstained with 4', 6'-diamidino-2-phenylindole (DAPI). Scale bars: 50 μm

Sweat glands mainly consist of secretory and ductal portions, the latter of which comprises epidermal, straight, and coiled ducts. The secretory portion consists of secretory luminal cells and encompassing myoepithelial cells, while the ductal portion consists of luminal cells and basal cells (Fig. 1). We first confirmed the localizations of stem cells by immunohistochemistry and histology using selectively expressed marker proteins. p63 is the dominate transcription factor of epidermal stem cell or progenitors, which involved in the balance between self-renewal and differentiation^29^. We found that p63 was strongly expressed in the epidermis and ducts of sweat gland, and moderately expressed in the secretory cells of sweat glands; similarly, the stem cell proliferation marker, Ki67, showed the same expression pattern (Fig. 2a).

Further, to determine accurately the location of SGCs in the palmo-plantar skin, expression profiles of markers for epidermis and sweat glands were examined in the epidermis, sweat ducts, and coiled structure compartments of sweat glands (Fig. 2b). CK14, a marker of cutaneous stem cells, was highly expressed in the epidermal basal layer and sweat gland duct portions, while weakly expressed in outer layers of secretory glands. CK5, another stem cell marker, was expressed in whole epidermis and the duct portions of sweat glands, but it was not observed in secretory sites. In contrast, CK10, the mature, differentiated marker, was detected in the spinous layer and granular layer, but not in the basal layer of epidermis and sweat glands. In a detailed analysis, each sweat gland compartment was studied using markers expressed exclusively by secretory luminal, and myoepithelial cells. SOX9, the major stem cell transcriptional regulator of the secretory niche signaling factor, was expressed only in the luminal layer of the sweat gland secretory region; in contrast, alpha-smooth muscle actin (αSMA) was expressed only in flattened small cells surrounding the sweat gland’s myoepithelial layers. The cells in the SGCs lose original epithelial markers and gain luminal epithelial markers, such as CK18 and CK19. Both of them, especially CK19, were detected in the secretory luminal layers, but not in the myoepithelial layers of sweat glands, according to the immunofluorescence (IF) staining and immunohistochemical assays (Supplementary Figure S1). The expression levels of each marker in epidermis, duct and secretory portion of sweat glands are summarized in Fig. 2b. Therefore, the chosen markers, αSMA, SOX9, CK18, and CK19, can be used as specific indicators to define isolated SGCs. Double-IF staining of secretory glands demonstrated that the expression of proteins related to sweat secretion and absorption (αATP, AQP5) were diminished in the αSMA-positive myoepithelial portions (Fig. 2c). This also included the glycosylated marker proteins of epithelial polarity (CEA), which was expressed within the secretory coils of sweat glands. These marker expressions can be used to indicate the SGCs and reconstructed sweat glands.

### Establishment of SGOs culture system

**Fig. 3:**
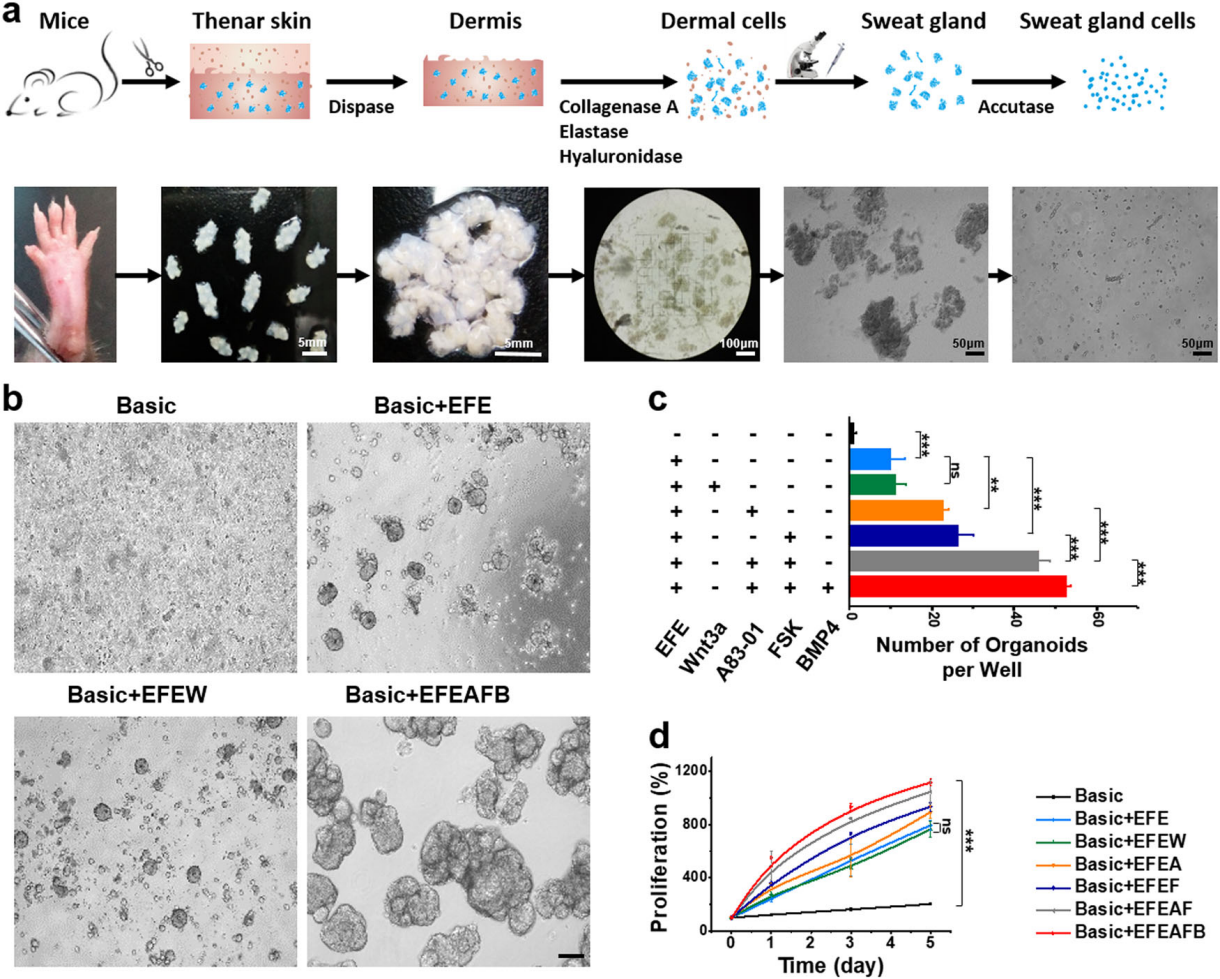
Efficient generation of SGOs from isolated sweat gland cells. **a** Schematic overview of isolation of the adult mouse SGCs. **b** Representative examples of 5-day-old organoids grown from SGCs at different culture conditions. **c** Quantification of organoid proliferation cultured in different defined mediums, as measured by the Alamar Blue cell viability assay. **d** Number of organoids formation derived from sweat gland cells in different culture conditions. **P < 0.01; ***P < 0.001. Scale bars: **b** 100 μm. SGO sweat gland organoid, SGC sweat gland cell

Dissociated epithelial cells in combination with partner mesenchymal cells can form organoids in 7–10 days^23,30^. Organoids can be repeatedly propagated through passaging in the presence of optimal niche factors. Both lumenal and myoepithelial portions contain stem cells in adult sweat glands, which should possess regenerative potential^31^. Sweat glands were mechanically isolated from a predigested and dissociated mouse thenar skin biopsy. Subsequently, isolated epithelia cells from sweat glands were embedded within Matrigel. Figure 3a showed the schematic of the sweat gland digestion and isolation into single cells. When skin samples were digested with the combination of collagenase A, elastase, and hyaluronidase at 37 °C for 1 h, most sweat glands were dissociated from adjacent connective tissue and released from peripheral tissues. These digested tissues not only expressed the sweat-gland-related markers, but also expressed some of the epidermis-related markers (Supplementary Figure S2). Following additional 15 min digestion with Accutase, the dissociated cells released from isolated sweat glands were plated into Matrigel to generate SGOs. The SGCs were originally maintained in the basic serum-free medium favored by several types of epithelia^21,24,32^. However, the efficiency of organoid formation was very low. In order to stimulate the gland stem/progenitor cell proliferation, several cell growth factors were added including epidermal growth factor (EGF)^33,34^, basic fibroblast growth factor (bFGF)^35^, and anhidrotic ectodermal dysplasias (EDAs)^36,37^, which were found previously to be involved in the development of skin. As expected, the basic medium with EGF, bFGF, and EDA (Basic + EFE) was essential for SGOs formation (Supplementary Figure S3a). More clone-like structures could be observed, and the number of organoids significantly increased (Fig. 3b, c). To further increase the generation efficiency of SGOs, several small molecules, which can activate or inhibit the key signaling pathways were tested. Wnt agonist, transforming growth factor beta (TGFβ) inhibitor, cyclic adenosine monophosphate (cAMP) agonist, and bone morphogenic protein (BMP) agonist were chosen because of the known relevance of these respective pathways in stem cells. In many tissues, Wnt pathway is a crucial signal in stem cell maintenance^19,38^. However, the SGOs formation did not change with or without the supplementation of Wnt agonist, Wnt3a. On the contrary, two small molecules, A83-01 (TGFβ signaling pathway Alk4/5/7 inhibitor) and Forskolin (FSK, cAMP pathway agonist), significantly improved the organoid formulating efficiency. Moreover, the combination of the two compounds synergistically increased the proliferation rate of SGCs. In addition, with the existence of the BMP agonist, BMP4, the gland-like organoids formation efficiency and cell proliferation could be further significantly improved (Fig. 3d). Therefore, the optimized culture condition, named as Basic + EFEAFB, included EGF, bFGF, EDA, A83-01, FSK and BMP4 as facultative culture components in the subsequent studies. Under this condition, the cultures developed in a stereotypical manner. After seeding, SGCs were able to generate SGOs with gland-like and colony-like structures. With the expansion of the cultures, organoids became more uniform. Cultures could be maintained for at least 2 months with weekly splitting rates of 1:5. After a longtime in culture, the SGOs consisted of several buddings, which surrounded a central lumen (Supplementary Figure S3b).

### SGOs maintained stemness traits

**Fig. 4:**
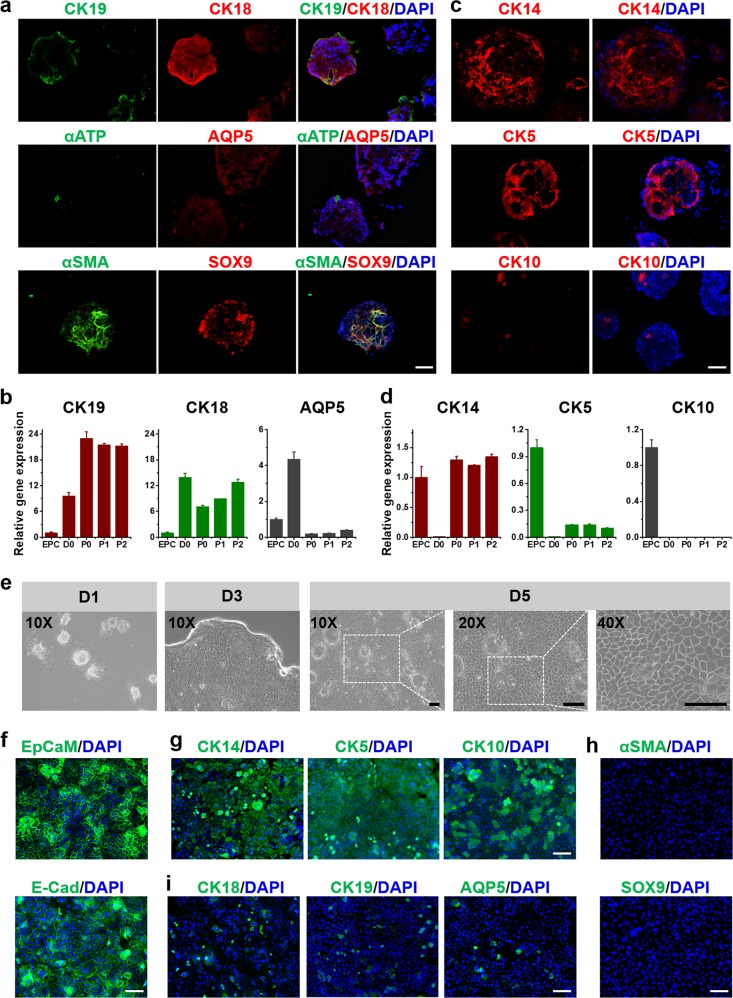
Marker expression of SGCs in organoids culture and monolayer culture. **a** IF staining indicated the expression levels of sweat-gland-specific markers (CK19, CK18, and αSMA), and functional markers (AQP5, αATP). **b** Gene expression of CK19, CK18, and AQP5 in sweat gland cells during the whole culture until to passage 2. The expression levels were normalized to that in epidermal cells (EPC). D0: pre-seed sweat gland cell; P0, P1, P2: the cells cultured in organoids at day 5 after passage 0, 1, and 2. **c** IF staining indicated the expression levels of epidermal cell markers, CK14, CK5, and CK10. **d** Gene expression of CK14, CK5, and CK10 in sweat gland cells. **e** Representative images of swear gland cells derived from organoids in monolayer culture. **f** IF staining of the epithelial cell adhesion molecule (EpCAM), and E-cadherin (E-Cad) in monolayer cultured swear gland cells. **g** Expression of epidermal cell markers, CK14, CK5 and CK10. **h** Expression of sweat gland stem cell markers, αSMA and SOX9. **i** Expression of sweat-gland-specific markers, CK18, CK19, and AQP5. Scale bars: 50 μm. SGC sweat gland cell, IF immunofluorescence

When adult mouse SGCs were cultured under 3D organotypic conditions, they generated gland-like aggregates. The hematoxylin-eosin (H&E) staining of these 3D organotypic cultures revealed many tubular-like structures with a centrally localized, hollow lumen (Supplementary Figure S4). Immunohistochemical analysis demonstrated that the cultured organoids expressed specific sweat gland luminal epithelial markers CK18 and/or CK19 (Fig. 4a). The sweat gland functional markers, AQP5 and αATP, were weakly expressed in these organoids. On the contrary, the stem cell marker, SOX9, was highly expressed in the epithelia, along with αSMA in the associated mesenchymal cells, indicating this organoid culture system provided a major environment for the stemness maintenance. These organoids had the potential to develop into sweat glands. Gene expression levels of CK18 and CK19 in the SGCs increased with organoid formation even after passaging, while AQP5 expression decreased compared with that in freshly isolated SGCs (Fig. 4b).

On the other hand, the epidermal markers, CK14 and CK5, were increasingly highly expressed in these cultured organoids (Fig. 4c). Especially the CK14 expression level in SGOs was comparable to that in freshly isolated epidermal cells (Fig. 4d). The gene expression of the differentiated marker CK10 in SGOs was much lower than that in epidermal cells. Therefore, a significant enrichment of stem cells with bipotential differentiation ability could be obtained in this organoid amplification culture system.

In order to verify the stemness of the organoids, the SGCs were further dissociated into single cells and cultured as monolayers. The cells grew clonogenically and formed “paving stone” structures with sharp edges on their borders such as that occurs in natural sweat glands (Fig. 4e). Further immunostaining results showed that epithelial cell adhesion molecule (EpCAM) was highly expressed evenly on the cell membrane (Fig. 4f). Interestingly, CK14 and CK5 expression increased in most of the 2D-cultured cells, with a few CK10+ epithelia cells appearing, verifying that SGCs differentiated epidermal cells began to appear that expressed CK10 (Fig. 4g). Meanwhile, almost no cells expressed αSMA and SOX9 (Fig. 4h), and only 8% of cells were CK18- or CK19-positive, which indicated that these cells had lost the potency to differentiate into SGCs (Fig. 4i). The comparison of these key indicators showed a significant difference between 3D organoids or 2D culture (Supplementary Figure S5). All the results illustrated the importance and necessity of organotypic culture for SGCs’ stem cell characteristic maintenance.

### SGOs could be induced into pseudostratified epidermis

**Fig. 5:**
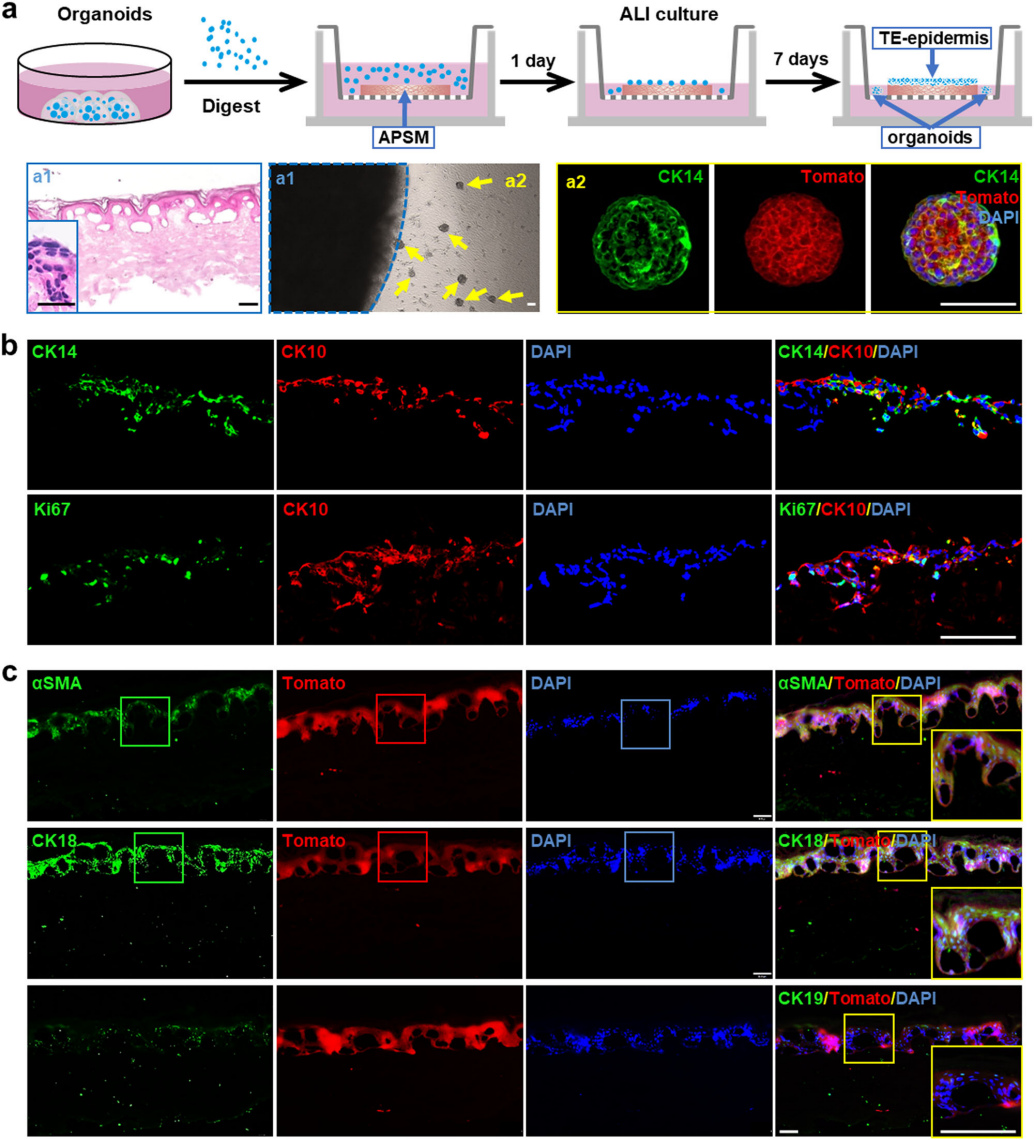
Establishment of tissue-engineered epidermis using SGCs derived from cultured organoids. **a** The schematic diagram of tissue-engineered epidermis using SGOs with acellular porcine skin matrix (APSM) under air−liquid interface (ALI) culture. The H&E of tissue-engineered epidermis (a1) and IF staining (a2) of epidermis organoids indicated the SGCs had the potential to differentiate and form the stratified epidermis. **b**, **c** IF staining of epidermal stem cell markers CK14, cell proliferation marker Ki67, and epidermal mature cell marker CK10 (**b**) and sweat gland stem cell marker αSMA, sweat-gland-specific markers CK18 and CK19 (**c**) in tissue-engineered epidermis. Scale bars: 50 μm. SGC sweat gland cell, SGO sweat gland organoid, IF immunofluorescence

To further verify the differentiation potential of the cultured SGOs, the SGCs with tdTomato tags were digested into single cells from SGOs and seeded onto acellular porcine skin matrix (APSM) (Fig. 5a). H&E staining showed that the SGCs could build up a confluent epithelial layer with a pseudostratified appearance after cultured on APSM for 7 days with air−liquid interface (ALI) culture (Fig. 5a(a1)). On the other hand, the SGCs formed spheres again within the medium without the ALI culture (Fig. 5a(a2)).

IF staining showed that in the basal layer of the pseudostratified epithelium, the cells expressed CK14, the marker of cutaneous stem cells, which also proved positive for Ki67. The stem cells derived from SGOs were able to be induced to differentiate into mature epidermal cells upon the basal layers, which gained CK10 expression (Fig. 5b). Interestingly, some of the cells in the pseudostratified epithelium also expressed αSMA, indicating they still had the precursor mesenchymal cells associated with sweat gland differentiation. More sweat gland markers, including CK18 and CK19, were expressed in the pseudostratified epithelium, illustrating they still maintained the characteristics of SGCs (Fig. 5c). These results demonstrated that the established SGOs can be used as seed cells to rebuild the epidermis with sweat gland formation potential in vitro.

### SGOs transplantation could promote the regeneration of epidermis and sweat gland in vivo

**Fig. 6:**
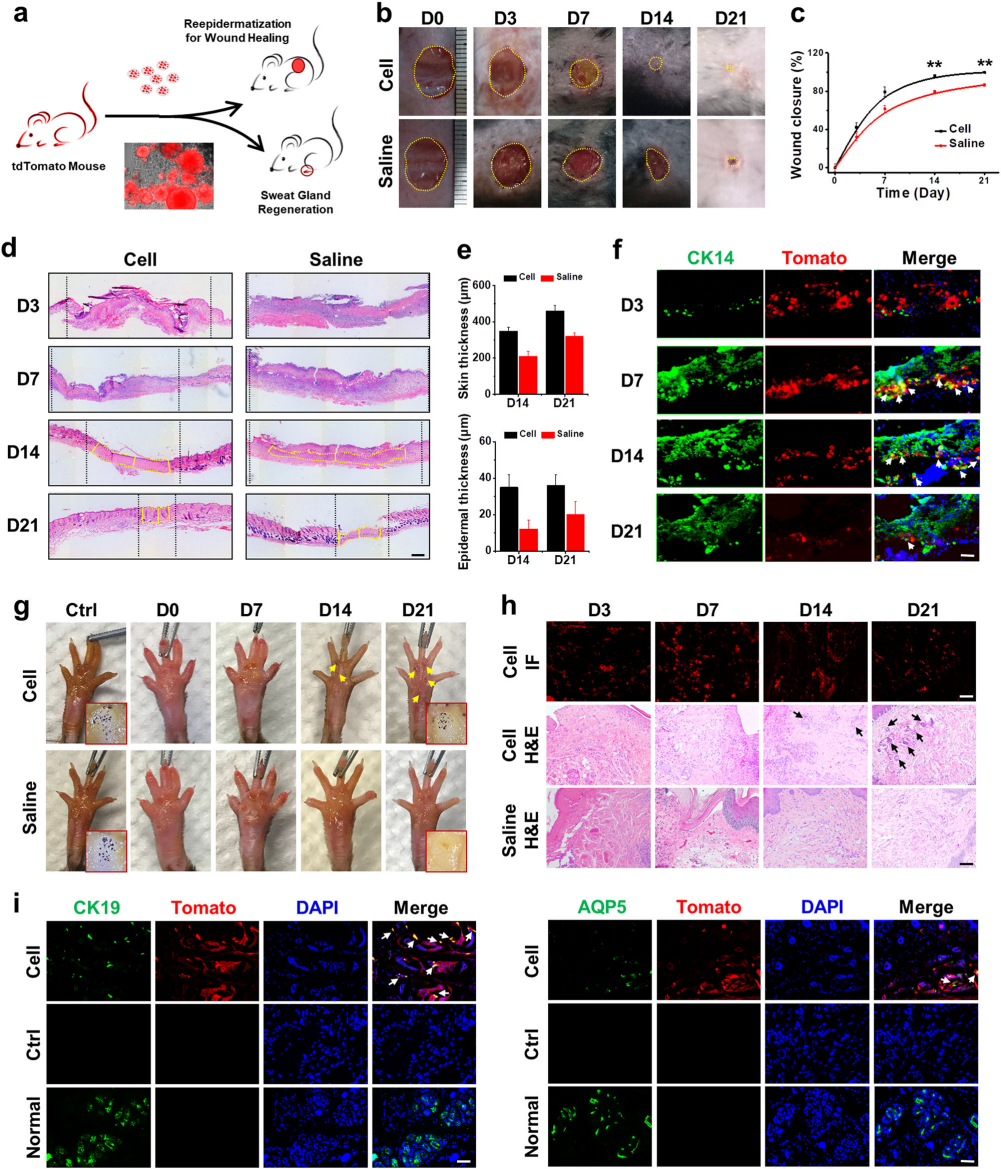
Transplantation of SGOs promoted wound healing and sweat gland regeneration in vivo. **a** The normal mice with wound or injured sweat glands were used to monitor the therapy effect of tdTomato-autologous SGCs isolated from syngeneic mice. **b** Representative images of wounds in mice of two test groups for 0–21 days. **c** Dermal wound closure (%) in the different treatment groups, and the wound area over time was measured as a percent of the original area. **d** Wound sections on 3, 7, 14, and 21 days of post transplantation (DPT) were stained with H&E for general observation of skin layers. **e** Skin thickness and epidermal thickness on 14 and 21 DPT in the different treatment groups. **f** IF staining showed the colocalization of the epidermal-specific marker, CK14, with transplanted tomato-sweat gland organoids at day 21 after transplantation in the cell-treated group. **g** Iodine/starch-based sweat test on paws of mice before injury and at days 0, 7, 14, and 21 after transplantation. Dark spots on foot paw pads were positive in cell-treated group. **h** The distribution of tdTomato-SGCs after transplantation, and the histology of sweat gland regeneration in mouse paws at days 3, 7, 14, and 21. **i** IF staining showed the colocalization of sweat-gland-specific marker, CK19, and functional marker, AQP5, with transplanted tdTomato-SGOs at day 21 after transplantation in the treated group. **P < 0.01. Scale bars: **d** 400 μm; **f**, **h**, **i** 100 μm. SGO sweat gland organoid, SGC sweat gland cell, IF immunofluorescence

As the SGCs derived from the established organtypic culture system still retain the ability to differentiate epidermis and sweat glands, these cells were used to treat full-thickness skin wound in mouse backs and scalded regions in mouse paws (Fig. 6a). The wound contraction, re-epithelialization and wound closure were analyzed in the mouse following transplantation. Remarkably, thick and well-formed granulation tissue was apparent in the transplantation group on day 7, indicating that SGCs could promote wound healing quickly (Fig. 6b–d). Results of quantitative analysis on skin and epidermal thickness revealed that both skin and epidermal thicknesses were higher than that of the saline group (Fig. 6e). The transplanted cells could be detected even on day 21 post wounding. Importantly, some of the tdTomato-positive cells coexpressed CK14, indicating that SGCs might participate in the epidermal regeneration (Fig. 6f).

To determine whether these cells in the SGOs could lead to sweat gland regeneration in vivo, the iodine/starch-based sweat test was performed on burned paws of mice. At a day before cell transplantation, the mice sweat gland injury model was established with their foot paw burnt at 65 °C for different times. The results confirmed that after 65 °C burn for 15 s, most of the secretory glands in deep paws were destroyed (Supplementary Figure S6). After the transplantation of SGCs for different times, starch was painted on the paws. Only mice with cell transplantation showed individual sweat glands (represented by black dots on foot pads), and the number increased within 10 min. However, no obvious black dot was observed in the saline group even after 20 min (Fig. 6g). Therefore, the SGCs treatment might repair the sweat glands in injured paws of mice. The SGCs could exist in paws as long as 21 days. Histological examination revealed that the damaged sweat glands were partially reconstructed with cells transplantation after 14 days. Injured paw skin, swollen with tiny bubbles, could be observed in control mice and almost no sweat glands could be regenerated even after 21 days (Fig. 6h). The tdTomato-labeled SGCs were also positive for CK19 and AQP5 in the reconstructed sweat glands in the hosts with cell transplantation (Fig. 6i). The results clearly demonstrated that the transplanted SGCs could participate in the regeneration of sweat glands. Given the above, these SGCs were able to participate in skin regeneration as well as sweat glands reconstruction.

## Discussion

The repair and regeneration of sweat gland structures and functions after severe burn injuries are tremendous challenges for clinical therapy. The main obstacle is to acquire appropriate and enough cells, which can be used to promote functional sweat gland reconstruction during the skin regeneration. Sweat gland stem/progenitor cells may not only repair destroyed skin structure, but also allow to recover the structure and function of injured sweat glands. Traditional models fail to reconstitute the in vivo cellular microenvironment and thus cannot maintain their functions. It is crucial to explore novel and suitable methods to isolate and culture SGCs in sufficient numbers and with strong functions^39^. A reproducible method of SGCs isolation and 3D organoid culture was developed in this study.

Recent reports have demonstrated the skin organoid were useful for studying mechanisms of hair follicle induction, evaluating drugs, and modeling skin diseases^27,40^. Klaka et al. established an organotypic sweat gland model with hanging drop system, and this model could display the physiological regulation of the sweat secretion process making it highly relevant for studying unknown mechanisms of sweating in vitro^41^. Liu et al. used 3D bioprinting matrices to guide the self-organized formation of sweat glands, and obtained a similar structure to natural developmental process^42^. Ryuichiro  et al. attempted to use the stem cells derived from sweat gland myoepithelial cells to generate spheroids. But the spheroids-forming efficiency was only ~2.5%^43^. There is no report to study the amplification of SGCs, due to the lack of specific culture medium for SGCs culture.

According to previous knowledge of organoid cultures in other tissues, we synchronously applied some growth factors and small molecules into sweat gland organotypic culture medium to create an inductive niche for SGCs amplification. The medium consisted of NIC, N-Ace, and B27, factors that have been reported useful for culturing epithelial cells^20,21^. After adding the common growth factors EGF and bFGF, and EDA, the medium efficiently improved the formation of SGOs. Further, A83-01, as a specific inhibitor of TGFβ signaling, could extend the cell culture time and enhance the colony-forming efficiency^23,44^. FSK has been reported to induce the proliferation of biliary duct cells in vivo^30^. And BMP4 can regulate the behavior of stem cells and affect their differentiation^45^. As a result, SGOs were generated effectively in a short time, and they remained in a state of rapid proliferation. We successfully established sweat gland organotypic cultures through optimization of culture conditions. Different morphological sweat gland structures such as clonal-like, duct-like, and gland-like structures were generated in sufficient quantities for consideration of cell therapies in vivo.

The generated organoids highly expressed SOX9 and αSMA, which is similar to the sweat gland in situ. Importantly, the stem-cell-related genes were maintained steadily and stably without any significant variation in levels from passaging, indicating that the cells in generated SGOs maintained stem cell characteristics and did not differentiate into sweat-gland-specific functional cells. A switch in cell identity and partial reprogramming might happen in organotypic culture, so that differentiated SGCs stopped to synthesize sweat-gland-related transcripts and started to express stem-cell-related genes. This finding supports that organoid culture system may provide an ideal niche-like microenvironment for stem cell enrichment and maintenance.

Previous evidences showed that SGCs have similar capability for wound healing as keratinocytes^13,46^. And the stem cell populations present in the sweat gland give rise to cells linked to keratinocyte stem cells^47^. In this study, to assess the bipotent properties of SGOs, we used the organoids as seeded cells to rebuild the tissue-engineered epidermis in vitro. A confluent epithelial layer with a stratified appearance was built up after ALI culture. Meanwhile, in the stratified epithelium, the cells not only expressed the keratinocyte markers but also expressed the sweat-gland-specific markers. Therefore, we may provide an ideal cell source for the regeneration of epidermis and sweat gland simultaneously.

The in vivo functional regeneration after transplantation is deemed to be the best judgment for evidence of translational clinical application. As expected, the cells derived from SGOs transplantation showed a substantial efficacy in cutaneous wound healing, and was able to achieve functional restoration of sweat glands. Meanwhile, the transplanted SGCs may participate in the entire process of wound healing and sweat gland regeneration. Combined with evidence from in vitro culture, these findings support that the SGOs culture provided a cell source for cell therapy in facilitating skin and sweat gland regeneration. The mechanisms involved in the improvement of wound healing and functional regeneration of sweat glands following cell implantation from SGOs are not fully understood. More probably, a significant number of cells can differentiate into cells of skin- or sweat gland lineages and facilitate skin with sweat gland reconstruction. And this phenomenon might partly be due to the changes in the microenvironment with the wound-healing process.

## Conclusion

The key finding in this study is that the optimized SGO cultures provide the appropriate cells in sufficient quantity for skin regeneration. These cells enhance healing and sweat gland repair during the regeneration process. The SGCs cultured under organoid-specific conditions were able to expand with enriched stem characteristics. In an ex vivo microenvironment mimicking skin, they were able to form pseudostratified epithelium with epidermal markers and also with sweat gland markers. After transplantation at an injured site, the SGOs were able to efficiently integrate into the tissue and enable skin regeneration. In summary, this study offers a new strategy for regenerating functional sweat-gland-like structures in deeply injured cutaneous wounds as well as facilitating wound healing.

## Experimental procedures

### Isolation of mouse SGCs

After euthanizing the mice and sterilizing the paw with 75% alcohol, the thenar skin specimens were excised and rinsed with phosphate-buffer saline (PBS), and the subcutaneous fat and blood vessels were carefully removed. Then these tissues were incubated in 2 mg/mL Dispase (17105041, Gibco, Life Technologies, CA) at 4 °C overnight. Following Dispase treatment, the epidermis and dermis were separated using forceps. The dermis tissues were minced into approximately 1.0 mm^3^ fragments with sharp scissors and digested with 2 U/mL Collagenase A (10103586001, Roche, USA), 0.5 U/mL Hyaluronidase (H3757, Sigma, USA), and 6 U/mL Elastase (E8140-1UN, Sigma, USA) at 37 °C for 1 h to dissociate the sweat glands. The samples obtained were centrifuged at 600 rpm for 3 min to remove the supernatant. The sweat glands were collected with a 100 μL pipettor by hand utilizing a phase contrast microscope (DMi1, Leica, Germany) to optimize visualization. The glands were further digested by Accutase (A1110501, Gibco, Life Technologies, CA) at 37 °C for 5 min to acquire the SGCs.

### Cell culture

For SGOs culture, 5×10^3^ SGCs per well were suspended in 1:1 of Advanced Dulbecco’s modified Eagle’s medium/F12 (adDF12) (12634010, Gibco, Life Technologies, CA) and Matrigel (354230, Corning, USA) in ultralow-adherence 24-well plates (3473, Corning, USA). After the Matrigel polymerization, complete sweat gland medium was prepared based on adDF12. The supplementary factors and molecules were listed in Supplementary Table S1.

For passaging, the SGOs were extracted by mechanically disrupting them with a pipet and cold media to depolymerize the Matrigel. The old Matrigel was washed away by spinning down at 600 rpm for 2 min; then the organoids were digested with Accutase at 37 °C for 5 min, and the fragments were replated in liquid Matrigel. Organoids were passaged every 7 days at a 1:5 ratio.

For monolayer cultures of SGCs, cells resuspended in sweat gland medium were seeded onto Matrigel-coated dishes.

### Reconstruction of tissue engineering epidermis in vitro

To study the differentiative potential of the SGOs, the organoids were used as seed cells to re-establish epidermis in vitro. The SGOs were digested into single cells with Accutase for 15 min. Then the SGCs were seeded on the basal membrane layer of APSM, and cultured for 1 day. Reconstruction of differentiated epidermis was performed at the ALI culture condition for 7 more days. The medium was changed everyday. The organization of reconstructed epidermis was examined by H&E and IF staining.

### In vivo examination of wound healing

Mouse backs were shaved then covered in a thin layer of Nair hair removal cream. A 1 × 1 cm^2^ of wound was created on the dorsal thoraco-lumber region of the mice subjected to ketamine anesthesia. The graft, containing 20 µL Matrigel with PBS (1:1) with or without 1000 SGOs, with about 4–5 million cells was applied directly to the wounds. Following surgery, the mice were wrapped with tegaderm (3 M, USA) to cover and protect the wound area. Subsequent measurements of wound area were taken at 3, 7, 14 and 21 days post transplantation. The results of wound measurements are expressed as percentage of wound area. Animals were sacrificed at different time points and wound tissue was excised and fixed for further histological evaluation and IF staining.

### In vivo starch-iodine assay for sweat gland formation

To determine the tissue-forming capability of SGOs, cells were transplanted into the burned paws of C57BL/6 mice. Mice were anesthetized with 2% isoflurane and then one hindpaw of each mouse was held in contact with the metal plate (65 °C) for 15 s. One day after the damage to the paw, ~100 SGOs in Matrigel were collected and transplanted under the damaged left paw skin. For each host, the control consisted of the right paw treated with blank Matrigel (i.e. without SGOs). Medical elastic bandages were used to cover the paw to keep the cells within the wounds. Then the mice were allowed to recover from anesthesia in their cages.

The iodine/starch sweat test was performed at 3, 7, 14 and 21 days after cell transplantation to evaluate sweat-gland-specific functions. For the test, animals were immobilized and the hind paws were painted with a solution of 2% (wt/v) iodine in ethanol. Once dry, a suspension of 10% (wt/v) starch in castor oil was painted on the surface of mouse thenar skin. Images of mouse paws were taken after the starchiodine assays to measure sweat secretion, which showed as dark spots. The sweat gland formation was also evaluated by histology and IF staining.

### Statistical analysis

All experiments were carried out in triplicate unless otherwise indicated. Error bars represent standard deviations. Data are presented as mean value ± SD from three independent measurements. Graphs were plotted using origin 9.0 software.

## Supplementary information

supporting information
