# Supplementary material for: Sweat gland organoids contribute to cutaneous wound healing and sweat gland regeneration
Source: Cell Death Dis. 2019 Mar 11;10(3):238. doi: 10.1038/s41419-019-1485-5 (PMC6411741; doi:10.1038/s41419-019-1485-5)
Supplement: Supplementary file 1 — supporting information [file 41419_2019_1485_MOESM1_ESM.doc]

**Sweat Gland Organoids Contribute to Cutaneous Wound Healing**

**and Sweat Gland Regeneration**

Jinmei Diao‡, Juan Liu‡, Shuyong Wang, Mingyang Chang, Xuan Wang, Baolin Guo, Qunfang Yu, Fang Yan, Yuxin Su, Yunfang Wang*

Stem Cell and Tissue Engineering Lab, Institute of Health Service and Transfusion Medicine, Beijing 100850, China.

**Corresponding Author**

* Address correspondence to: Dr. Y. Wang, Stem Cell and Tissue Engineering Lab, Institute of Health Service and Transfusion Medicine. 27, Taiping Road, Haidian District, Beijing, 100850, P.R. China. Tel: +86-10-66931545; Email: wangyf1972@gmail.com.

‡ J.D. and J.L. contributed equally to this work.

**Supplementary Experimental Procedures**

**Animals**

C57BL/6 mice (8 weeks old) were obtained from Beijing Vital River Laboratory Animal Technology Company Co., Ltd (Beijing, China). C57BL/6 mice with red fluorescent protein (tdTomato) (6 weeks old) were purchased from Shanghai Southern Model Biological Research Center (Shanghai, China). All of the mice were maintained in the Laboratory Animal Center of the Academy of Military Medical Sciences. All animal procedures were performed according to protocols approved by the Institutional Animal Care and Use Committee at Beijing Institute of Health Service and Transfusion Medicine.

**Cell Proliferation Assay**

To examine the proliferation of SGOs cultured in different media, the Alamar Blue assay (Cell Viability Reagent, Invitrogen, Life Technologies, USA) was performed after 1, 2, 3, 4, and 5 days of cultivation of the cells in organoid cultures in different conditions, according to the manufacturer’s instructions. After 2 h of incubation with Alamar Blue reagent, the fluorescent intensity of samples was measured using the microplate reader (EnSight, PerkinElmer, USA) at wavelengths of 530 nm for excitation and 590 nm for emission.

**Histological, Immunohistochemical (IHC) Analysis, and Immunofluorescence (IF) Staining**

For IHC analysis of mouse paw skin, SGOs, and skin tissues, the samples were rinsed with PBS and fixed in 4 % paraformaldehyde for 1 h, and embedded in paraffin. Sections at 5 μm thickness were prepared for H&E, and IHC staining. For IF staining of mouse pawskin, SGOs, tissue engineered epidermis, and skin tissues, the samples were then dehydrated in 20 % sucrose solution for 24 h, and embedded in OCT. Frozen sections with 10 μm were prepared. Briefly, slides were rehydrated and stained with primary antibodies (Supplementary Table S2). Subsequently, the samples were incubated with the universal secondary antibody (Invitrogen, Life Technologies, USA) and VECTASTAIN Elite ABC reagent (PK6200, Vector, Germany), reacted with ImmPACT DAB enzyme substrate (SK4800, Vector, Germany), and counterstained with hematoxylin (H3404, Vector, Germany). Alternatively, the fluorescent secondary antibodies were used to visualize the stained sections, and DAPI was used as a nuclear counter staining. Finally, H&E, IHC and fluorescent images were taken with the Vectra® 3 automated quantitative pathology imaging system (PerkinElmer, USA) and analyzed with ImageJ software. For the IF staining of 2D cultured cells, cells were fixed and stained with primary antibodies (Supplementary Table S2). Fluorescent secondary antibodies were added for visualizing. Cells were counterstained with DAPI for visualization of cell nuclei and observed using a confocal microscope (LSM880, ZEISS, Germany).

**qRT-PCR Analysis**

Total RNA was isolated using the RNeasy Mini Kit (Qiangen, Germany), after which cDNA was synthesized with reverse transcriptase (ReverTra Ace® qPCR RT Master Mix, Toyobo) according to the manufacturer’s instructions. The qRT-PCR was performed with SYBR green master mix (TOYOBO) on a Bio-Rad iQ5 Real-Time PCR detection system. Data were collected using Bio-Rad CFX Manager software, and the expression of genes within a sample was normalized to GAPDH expression by the 2^-ΔΔCt^ method. The primers used in this study are listed in Supplementary Table S3.


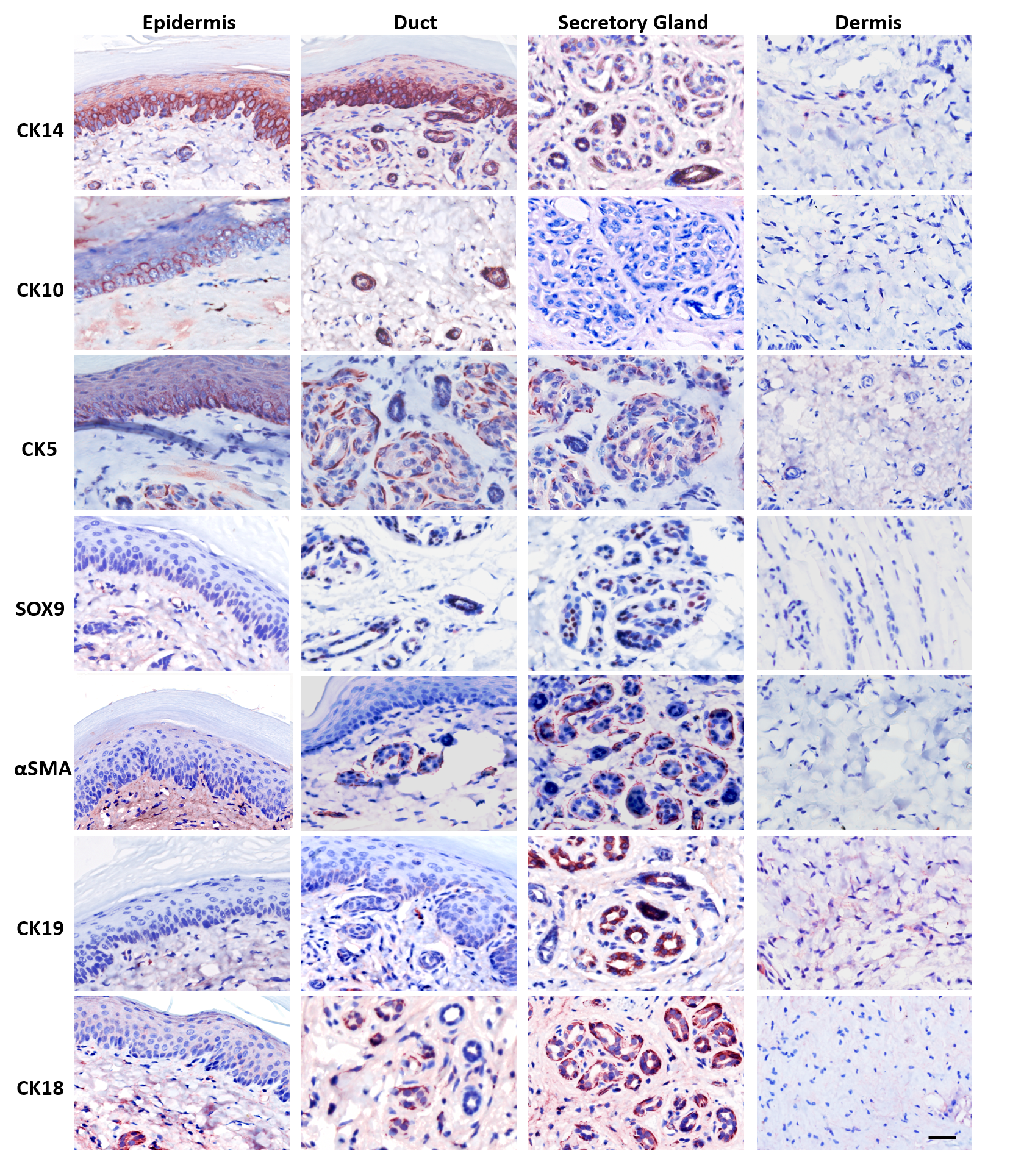


**Supplementary Figure S1** IHC staining reveals the different expression of markers specific between sweat gland and epidermis in mouse paw skin. CK14 was highly expressed in epidermal basal layers and sweat gland ducts while weakly expressed in myoepithelial cells of the sweat gland secretory portions. CK10 was highly expressed in the basal layer of the epidermis and was observed in sweat gland ducts. CK5 was expressed in both epidermis and sweat gland duct and secretory portions. SOX9 and αSMA were expressed only in sweat gland duct and secretory portions while not was expressed in epidermis. Furthermore, CK19 and CK18 were only expressed in sweat gland secretory portions. Almost no markers were expressed in skin dermis. Nuclei were stained with hematoxylin. Scale bar: 50 μm.


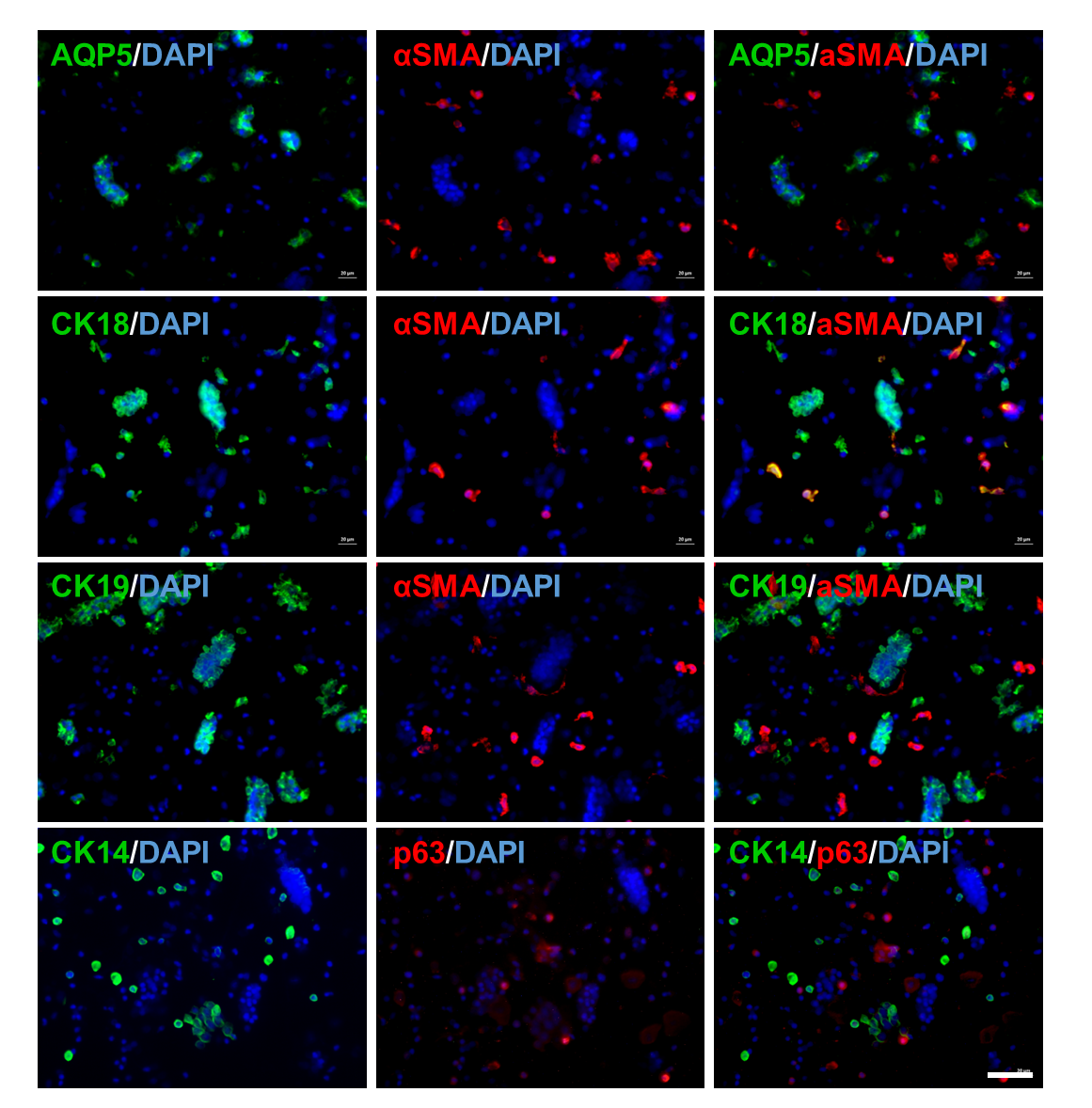


**Supplementary Figure S2** IF staining of the expression of relevant biomarkers in isolated SGCs. Nuclei were counter stained with DAPI. Scale bar: 50 μm.


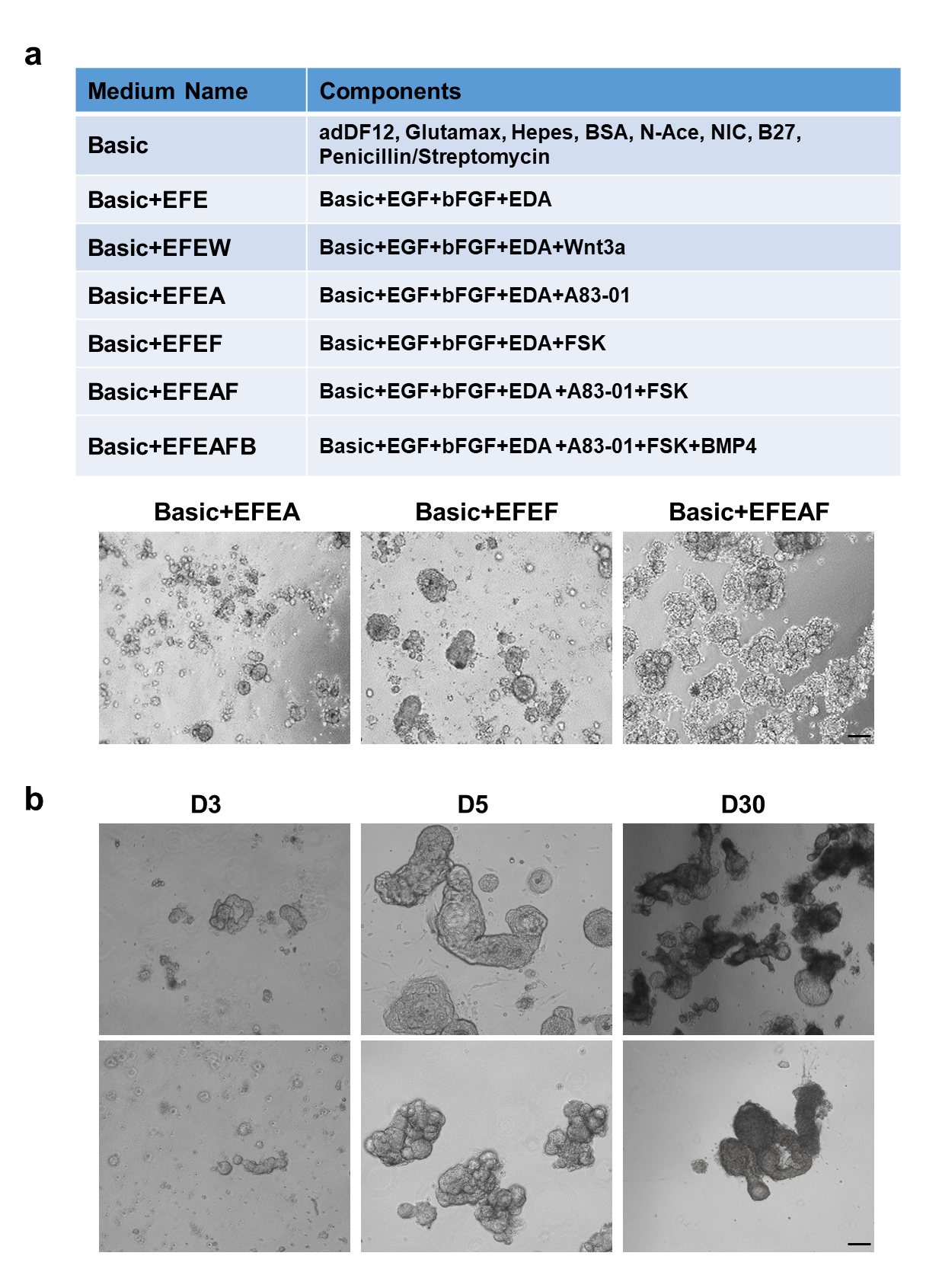


**Supplementary Figure S3** Generation of SGOs derived from mouse SGCs. **A** Established an optimized 3D culture system for SGOs. **B** Bright field images of SGOs cultured for different lengths of time (D3, D5, D30) with the optimized culture conditions (Basic+EFEAFB). Scale bar:100 μm.

**
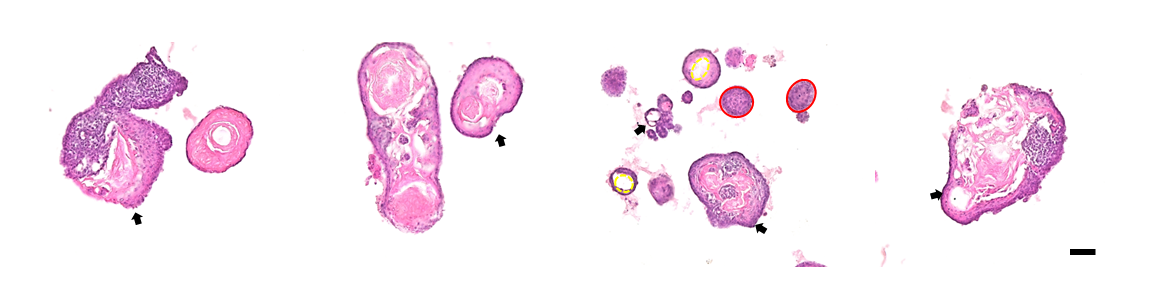
**

**Supplementary Figure S4** Morphology of generated SGOs. H&E staining showed that mouse SGCs formed different structures containing colony-like (red solid line), duct-like (yellow dash line) and gland-like (black arrow) phenotypic traits. Scale bar: 50 μm.


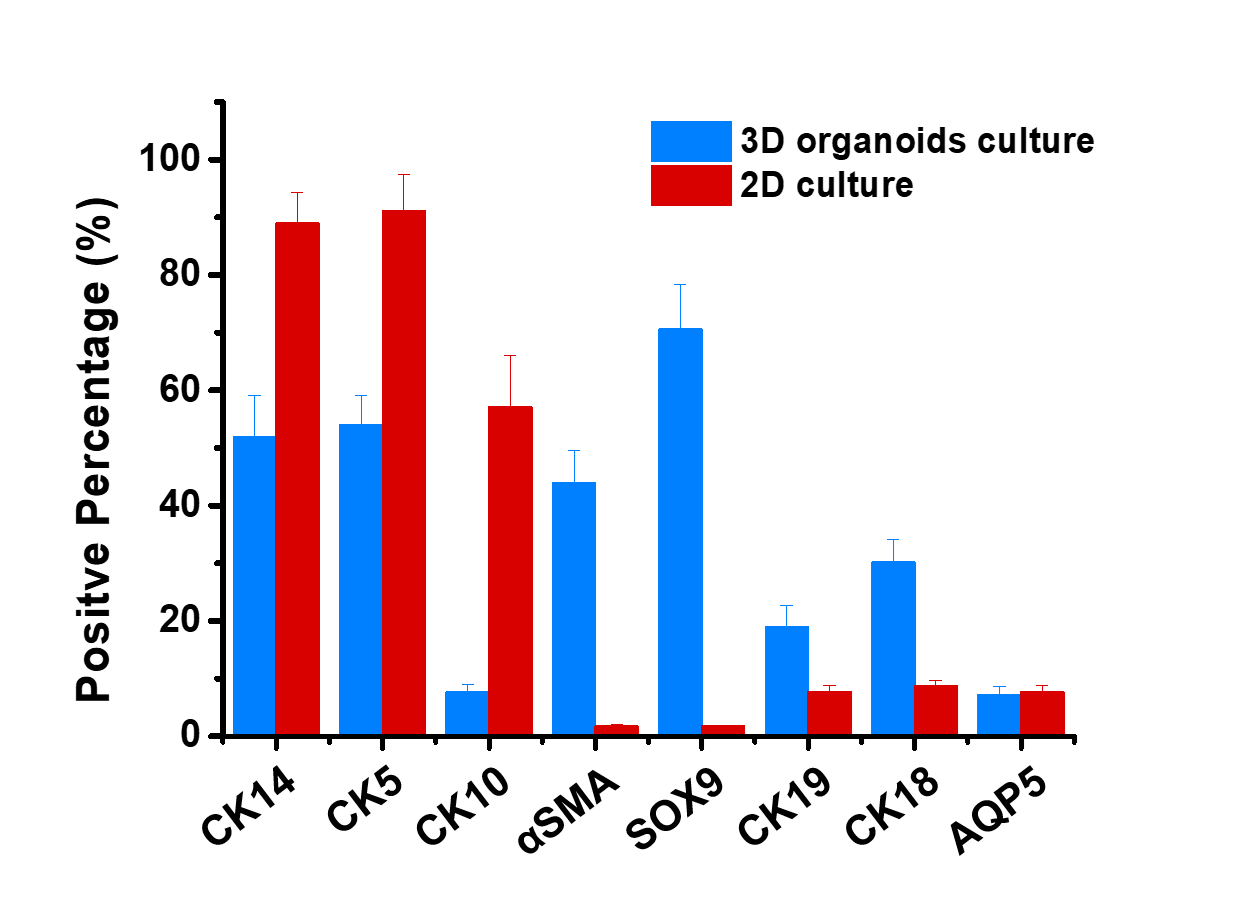


**Supplementary Figure S5** Quantification of different markers expression. Expression levels of different markers in SGCs under 3D organoids culture and 2D culture quantified by ImageJ.


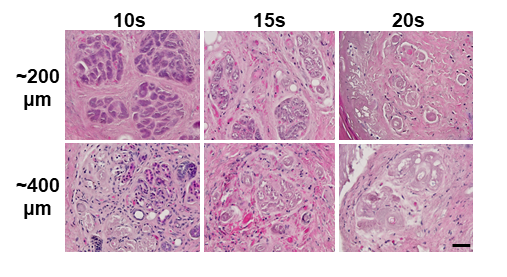


**Supplementary Figure S6** Establishment of sweat gland injury model. H&E staining showed the extent of sweat gland injury at different depths of skin (~200 μm and ~400 μm) at 65 ^o^C for 10 s, 15 s and 20 s. Scale bar: 50 μm.

**Supplementary Table S1.** Overview of medium components.

| **Regent** | Concentration | **Company** |
| --- | --- | --- |
| Advanced DMEM/F12 | 1 X | Gibco |
| BSA | 0.1 % | Gibco |
| Glutamax | 100 X | Gibco |
| Hepes | 100 X | Gibco |
| B27 | 50 X | Gibco |
| N-Ace | 1.25 μM | Sigma |
| NIC | 10 mM | Sigma |
| Penicillin/Streptomycin | 100 U/ml | Sigma |
| EGF | 50 ng/ml | R&D |
| bFGF | 20 ng/ml | R&D |
| EDA | 20 ng/ml | R&D |
| Wnt3a | 20 ng/ml | R&D |
| BMP4 | 20 ng/ml | R&D |
| A83-01 | 1 μM | Selleck |
| FSK | 10 μM | Selleck |

**Supplementary Table S2.** Antibodies used in this study.

| **Primary Antibody** | **Company** | **Product Code** | **IgG Species** | **Dilution** |
| --- | --- | --- | --- | --- |
| αΑTP | Abcam | ab2872 | Mouse IgG1 | 1:100 |
| αSMA | Abcam | ab28052 | Mouse IgM | 1:100 |
| AQP5 | Atlas | R91463 | Rabbit | 1:1000 |
| CEA | Abcam | ab134074 | Rabbit | 1:100 |
| CK5 | Abcam | ab53121 | Rabbit | 1:500 |
| CK10 | Abcam | ab9026 | Mouse IgG1 | 1:100 |
| CK14 | Abcam . | ab181595 | Rabbit | 1:1000 |
| CK18 | Abcam | ab668 | Mouse IgG | 1:100 |
| CK19 | Abcam | ab52625 | Rabbit | 1:200 |
| E-cadherin | Abcam | ab40772 | Rabbit | 1:100 |
| EpCAM | Abcam | ab71916 | Rabbit | 1:100 |
| Ki67 | Abcam | ab15580 | Rabbit | 1:100 |
| p63 | Abcam | ab735 | Mouse IgG2a | 1:50 |
| SOX9 | Abcam | ab76997 | Rabbit | 1:100 |

| **Secondary Antibody** | **Company** | **Product Code** | **Dilution** |
| --- | --- | --- | --- |
| Alexa Fluor® 488 Goat Anti-Mouse IgG2a (γ2a) | Invitrogen | A21131 | 1:400 |
| Alexa Fluor® 568 Goat Anti-Mouse IgG1 (γ1) | Invitrogen | A21124 | 1:400 |
| Alexa Fluor® 647 Goat Anti-Rabbit IgG (H+L) | Invitrogen | A11031 | 1:400 |

**Supplementary Table S3.** Primers used in this study

| **Gene** | **Forward** | **Reverse** |
| --- | --- | --- |
| AQP5 | AGAAGGAGGTGTGTTCAGTTGC | GCCAGAGTAATGGCCGGAT |
| CK5 | TCTGCCATCACCCCATCTGT | CCTCCGCCAGAACTGTAGGA |
| CK10 | CGAAGAGCTGGCCTACCTAAA | GGGCAGCGTTCATTTCCAC |
| CK14 | AGCGGCAAGAGTGAGATTTCT | CCTCCAGGTTATTCTCCAGGG |
| CK18 | ACTCCGCAAGGTGGTAGATGA | TCCACTTCCACAGTCAATCCA |
| CK19 | GGGGGTTCAGTACGCATTGG | GAGGACGAGGTCACGAAGC |
| GADPH | AGGTCGGTGTGAACGGATTTG | TGTAGACCATGTAGTTGAGGTCAA |
